# Personal Health Management (PHM): Singapore’s national strategy to activate and empower patients and care givers through innovative personal health technologies

**Published:** 2012-06-15

**Authors:** Tikki Gee

**Affiliations:** MOH Holdings, Singapore

**Keywords:** self management, strategy, policy, mhealth, telehealth

## Abstract

**Introduction:**

In the next two decades, Singapore will face a near-perfect demographic and chronic disease-burden “storm”. Rising public expectations of healthcare services, inflationary cost pressures and continuous resource scarcity add to the challenges the system faces. Singapore’s Ministry of Health’s (MOH) response to these impending challenges has been swift and reforms are under way that will lead to new models of care, integrated care delivery capabilities as well as increased capacity (through development of primary care and new facilities) in light of growing demands. The national Personal Health Management (PHM) strategy adds another dimension to Singapore’s national reforms, which is to leverage on one of the greatest untapped resources of healthcare: people, their families and communities.

**Aims and objectives:**

At the core of PHM is self-management and Singapore’s continuous promotion of personal responsibility. To support self-management, there is a need to provide patients/people with access to timely, actionable health information—key ingredients of empowerment that leads to greater self-efficacy. Instead of the traditional approach of developing a “static” patient portal, Singapore is taking a unique approach of developing an “open” health technology platform capable of catering to diverse stakeholder needs, and one that allow healthcare providers, enterprises, interest groups to create and build web, mobile applications and interactive content on a common platform to support existing and new healthcare programmes and services. At the crux of the platform is personal health record which is a subset of the just launched, national electronic health record (NEHR) that provides a longitudinal view of the person’s health information generated through life-time encounters at various care settings. The development of a national demonstrator PHM project is underway, slated for launch in early Q2 2012 with participation of two regional healthcare providers aimed at providing self-management technology tools (web and mobile) for low-medium risk diabetic patients. This paper/presentation aims to outline and share Singapore’s approach to empowering patients through the national strategy, barriers and its implementation thus far and roadmap going forward.

**Results:**

It is too early to be able to provide measureable outcomes in particular, clinical outcomes until steady-state is achieved beyond 2012. PHM is a large transformational project where the challenge goes beyond just the implementation of the technology. This is largely due to how the healthcare system is structured and financed in Singapore. The development of the national strategy has been a significant milestone; in that it has galvanised an otherwise disparate approach to self-management that will result in siloed patient information and duplication of efforts. The strategy has garnered senior leadership support from the ministry and stakeholder commitment to collaborate on the platform was a major step forward.

**Conclusion:**

The PHM strategy is the start of an exciting journey to enable a transformation of Singapore’s healthcare system that truly puts the person in the driver’s seat of their own health. The realisation of the PHM vision will take 10 years and development will be in 3 phases starting in 2011. The successful execution of the strategy relies on close coordination and cooperation among its stakeholders. The proposed “open platform” approach recognises that there will not be a one-size-fit-all solution and that diversity will be an added strength.

